# Understanding the need for digital twins’ data in patient advocacy and forecasting oncology

**DOI:** 10.3389/frai.2023.1260361

**Published:** 2023-11-10

**Authors:** Hung-Ching Chang, Antony M. Gitau, Siri Kothapalli, Danny R. Welch, Mihaela E. Sardiu, Matthew D. McCoy

**Affiliations:** ^1^Department of Biostatistics, University of Pittsburgh, Pittsburgh, PA, United States; ^2^Department of Electrical and Electronics Engineering, Kenyatta University, Nairobi, Kenya; ^3^Department of Engineering and Computer Science, Baylor University, Waco, TX, United States; ^4^Department of Cancer Biology, University of Kansas Medical Center, Kansas City, KS, United States; ^5^The University of Kansas Cancer Center, Kansas City, KS, United States; ^6^Department of Biostatistics and Data Science, University of Kansas Medical Center, Kansas City, KS, United States; ^7^Kansas Institute for Precision Medicine, University of Kansas Medical Center, Kansas City, KS, United States; ^8^Innovation Center for Biomedical Informatics, Department of Oncology, Georgetown University Medical Center, Washington, DC, United States; ^9^Lombardi Comprehensive Cancer Center, Washington, DC, United States

**Keywords:** digital twins, cancer, precision medicine, omics, data integration

## Abstract

Digital twins are made of a real-world component where data is measured and a virtual component where those measurements are used to parameterize computational models. There is growing interest in applying digital twins-based approaches to optimize personalized treatment plans and improve health outcomes. The integration of artificial intelligence is critical in this process, as it enables the development of sophisticated disease models that can accurately predict patient response to therapeutic interventions. There is a unique and equally important application of AI to the real-world component of a digital twin when it is applied to medical interventions. The patient can only be treated once, and therefore, we must turn to the experience and outcomes of previously treated patients for validation and optimization of the computational predictions. The physical component of a digital twins instead must utilize a compilation of available data from previously treated cancer patients whose characteristics (genetics, tumor type, lifestyle, etc.) closely parallel those of a newly diagnosed cancer patient for the purpose of predicting outcomes, stratifying treatment options, predicting responses to treatment and/or adverse events. These tasks include the development of robust data collection methods, ensuring data availability, creating precise and dependable models, and establishing ethical guidelines for the use and sharing of data. To successfully implement digital twin technology in clinical care, it is crucial to gather data that accurately reflects the variety of diseases and the diversity of the population.

## 1. Introduction

Digital Twins (DT) have gained widespread adoption as a crucial component of the industrial engineering approach to optimize manufacturing processes and drive advancements in modern industrial design. However, the transformative potential of DT extends beyond the industrial realm and finds numerous impactful applications in healthcare (Madhavan et al., 2021; Stahlberg et al., 2022). In particular, DT have emerged as powerful tools in precision medicine, clinical trials, and drug development (Haleem et al., 2023; Sun et al., 2023). A notable example is Unlearn. AI, a pioneering startup that secured an impressive $17 million in funding to develop DT specifically for trials. In a groundbreaking study by Venkatesh et al. (2022), Unlearn. AI predicted that Health Digital Twins (HDT) could potentially reduce clinical trial expenses by a staggering 25%. This groundbreaking finding underscores the immense value and potential of DT in revolutionizing the healthcare landscape and highlights their ability to drive substantial cost savings while enhancing patient outcomes.

In its most general form, a DT consists of a real-world object and its virtual counterpart, which work together to enhance each other through an iterative process. Data is collected under different conditions to refine the computational simulations, and the virtual representation is used to predict how to optimize the real-world system. By testing these predictions, we can identify their success and failure, and further improve the virtual model. As a result, through each DT iteration, the real-world system is continuously updated and improved.

In order to avoid altering the state of a real-world system, successful DT applications often rely on having identical copies of the real-world counterpart available for testing the predicted response. This allows for testing multiple, potentially destructive conditions, and, unfortunately, does not apply to HDT. In the absence of identical copies of an individual, we must look to other patients when training predictive models and evaluating the predicted outcomes of various therapeutic interventions. These patients are closely matched with respect to their biological characteristics, and longitudinal data detailing their intervention and response, and the outcome can serve to validate model predictions.

The incorporation of artificial intelligence (AI) plays a pivotal role in the realm of DT and can be dissected into three key components: (1) identify patient data from individuals that closely mimic the biological system of the target patient to evaluate predicted outcomes; (2) predict the patient outcome to optimize an individualized therapeutic approach and reduce the cost associated with healthcare interventions; (3) unravel the underlying biological mechanisms that drive diseases. It is worth noting that components (2) and (3) would greatly benefit from an efficient method for identifying real-world DT, as the increased information on patient disease and response would expedite the development of disease models and drive further understanding disease mechanisms.

**Figure 1:**
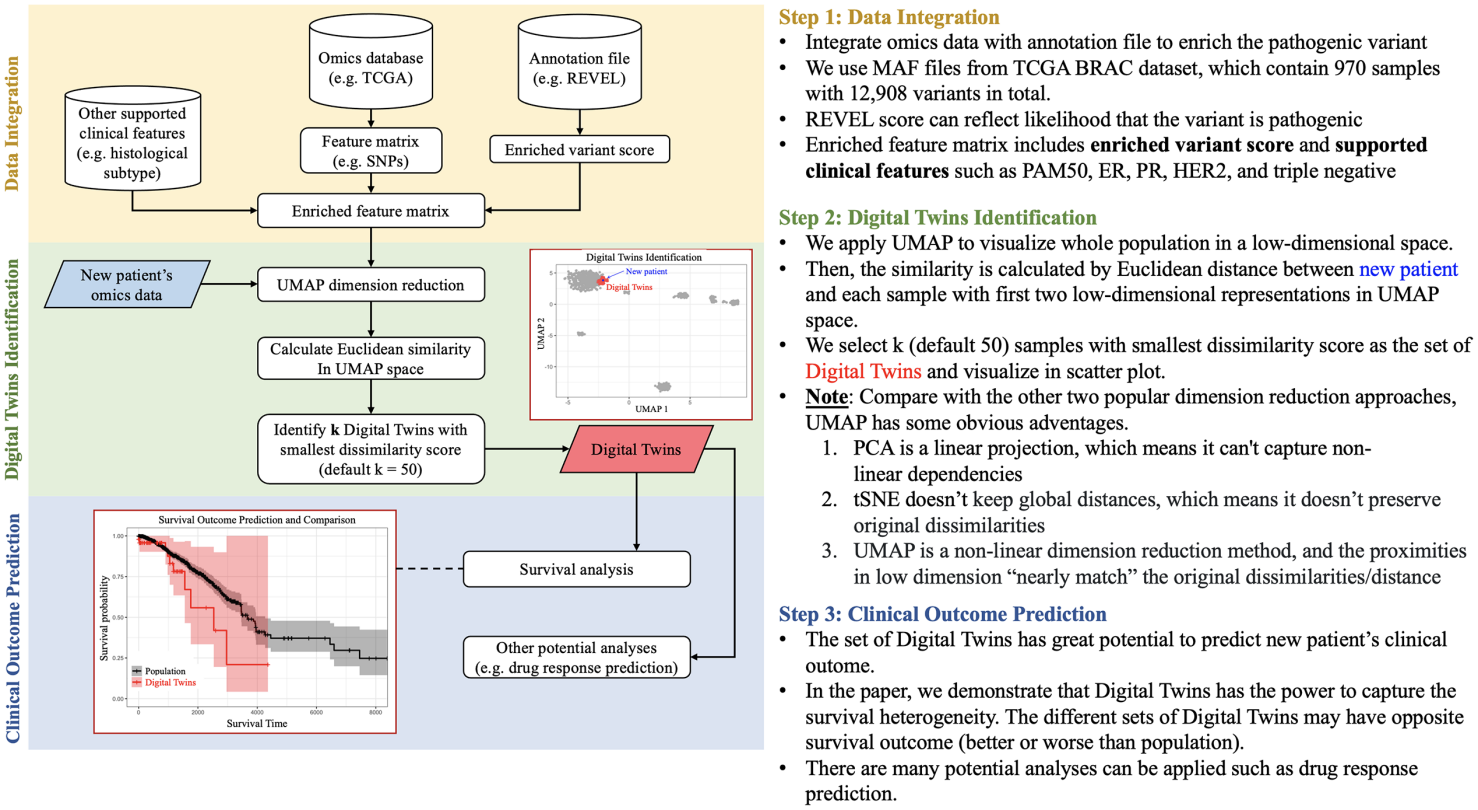
Pipeline of patient DT identification. The pipeline is divided into three stages: (i) Data Integration for integrating enriched omics and clinical data, (ii) Digital Twins identification based on Euclidean distance in UMAP space, and (iii) Prediction of clinical outcomes for better patient stratification.

To collect real-world datasets that closely align with an individual patient’s DT, we present a novel approach that capitalizes on the public availability of high throughput “omics” data and represents an early iteration of a workflow for identifying matched datasets to incorporate into patient DT. In the 21st century, many studies (McClellan and King, 2010; Tam et al., 2019) propose that human disease can be characterized by marked genetic heterogeneity. While some studies apply clinical feature maps to represent a real-world patient virtually (Venkatesh et al., 2022), we propose a pioneering concept that characterizes clinical and molecular features (Figure 1) from The Cancer Genome Atlas (Weinstein et al., 2013) and identifies a subset of individuals to incorporate into a given patient’s DT. The outcomes and experience of this subset of “like” individuals can then be used to evaluate the robustness of the computational predictions.

Our methodology begins by constructing a low-dimensional embedding of clinical and molecular features, enriched by an external annotation database. This low-dimensional feature map serves as the foundation for identifying a subset of individuals most representative of the DT through Euclidean similarity, and the results are visually presented using UMAP. Consequently, these embeddings offer a better representation of each patient by projecting those with similar genetic patterns and clinical features to the same region of the plot. The selected DT have the potential to predict the survival outcomes of new patients and even forecast drug responses. Furthermore, this pipeline can be extended to incorporate multi-omics data and environmental factors, enhancing its adaptability and comprehensive analytical capabilities.

## 2. Challenges to data collection

When collecting biological or clinical data for DT, there are various challenges that need to be addressed to ensure accurate and valuable representations of individuals. DT data is collected at various scales, capturing different types of information related to lifestyle, clinical and routine diagnostic data, as well as research data. For instance, they can gather data on individual lifestyle factors like physical activity, sleep patterns, nutrition, and stress levels, using wearable devices, mobile apps, or self-reporting. Additionally, they can collect clinical data on medication usage, laboratory test results, and vital signs, which is typically sourced from electronic health records (EHRs), health monitoring devices, or patient self-reporting. Processing and analyzing large amounts of data in digital twin computational models requires considerable computational power. To effectively handle the computational demands of these models, it is crucial to have a high-performance computing infrastructure, consisting of powerful processors, memory, and storage systems.

In addition to the many challenges associated with the data collection, the fact that the data comes from human patients presents additional considerations. These include data accessibility, data quality, standardization, data complexity, and ethical and privacy considerations. For example, getting the required biological data can be difficult because of privacy regulations, restricted entry to medical records or research databases, and the need for agreement from participants. It is essential to access diverse and representative datasets to create thorough and widely applicable DT. Furthermore, collecting and utilizing biological data for DT raises ethical and privacy concerns, such as informed consent, data anonymization, and data sharing. Ensuring compliance with ethical guidelines and implementing robust data security measures are essential to protect individual privacy and maintain public trust.

To tackle these obstacles, researchers, clinicians, data scientists, and policymakers need to work together. They must establish consistent protocols, create innovative analytics tools, and navigate ethical concerns. By overcoming these challenges, we can achieve more precise and trustworthy DT to benefit personalized medicine and healthcare.

## 3. Modeling for breast cancer

When applied to cancer patients, DT offer a forward-thinking approach to healthcare by creating virtual representations of patients. These representations use mathematical and computational models to simulate personalized diagnosis and treatment options and are increasingly integrating AI in several ways. In addition to directly learning the relationships between high throughput omics and clinical datasets and outcomes, it also serves a role in mapping patient data directly to model parameters. By utilizing DT, healthcare professionals can analyze the potential effects of different treatment approaches, leading to more informed decision-making and personalized patient care (Hernandez-Boussard et al., 2021).

Applying the digital twin paradigm to human subjects creates yet another application of AI, identifying previously treated patients that can bolster the data in the real-world component of the DT. It is impossible to test and validate multiple therapeutic interventions, and data on how patients who are matched on both their individual and tumor biologies must be used to evaluate the robustness of the virtual component of the DT.

Shown in Figure 1, a workflow was developed as part of a community hackathon event with the goal of utilizing public data (specifically, the BRCA (Breast Invasive Carcinoma) dataset from The Cancer Genome Atlas (TCGA), which provides genomic, histological, and clinical data on breast cancer patients) to build a real-world cohort of patients from a query patient. To identify additional patient data as input to a patient’s DT, the data analysis process is divided into three stages: (a) Data Integration, (b) DT identification, and (c) Prediction of Clinical Outcomes. Our objective is to conduct an integrative analysis of BRCA data utilizing omics techniques, such as enriched variant scoring (Ioannidis et al., 2016), and clinical features, such as histological subtypes for outcome prediction and DT identification (Liu et al., 2018; Thennavan et al., 2021). The BRCA dataset from TCGA consists of 970 samples and 12,908 variants. We utilize annotation files from REVEL to enhance our analysis by assessing the likelihood of a variant being pathogenic. Our final feature matrix is enriched with variant scores and supported clinical features such as PAM50, ER, PR, HER2, and triple negative subtyping data. We then utilized the UMAP clustering approach to identify real world examples that closely matched data from the input DT. This involved visualizing the entire population and calculating similarity by measuring the Euclidean distance between new patients and each sample using the first two low-dimensional representations in UMAP space. We selected k (default 50) samples with the smallest dissimilarity score as the set of DT and visualized them in the scatter plot. Through our research, we have discovered that DT can capture survival heterogeneity, making them a valuable resource for predicting the clinical outcome of new patients. It is important to note that the various sets of DT may have different survival outcomes, either better or worse than the overall population. Our survival data is obtained from UCSC Xena (Goldman et al., 2020).

Our approach can benefit from incorporating various other data types and computational modelling, including data that can be utilized for drug response prediction and prioritization of therapeutic options (McCoy and Madhavan, 2018; Kumar et al., 2019; Adam et al., 2020; Chang et al., 2020; Baptista et al., 2021; McCoy et al., 2021; Thiemeyer et al., 2021; Babbitt et al., 2022; Wang et al., 2022). DT can greatly aid breast cancer research by allowing for better patient stratification, improved treatment decision-making, and faster drug development. This technique has the capability to revolutionize breast cancer management by promoting personalized medicine and optimizing clinical practices.

## 4. Discussion

Artificial intelligence will play a crucial role in deploying DT to cancer patients. Aside from the direct application as models that predict the outcomes of different therapeutic interventions, they will also map patient data to parameters in mechanistic models of disease progression. However, applying AI to supplement the real-world component of the DT is equally essential. The progression and outcomes of the computational models of divergent intervention strategies can be validated by directly comparing patient trajectories and experiences. Also, the subset of patients that most closely match the real-world component of the DT can be used to evaluate the robustness of model predictions by providing additional inputs. For any given model prediction, convergence of its output for the subset of real-world counterparts can boost confidence in the predicted outcomes.

The utilization of DT has the potential to greatly benefit patients and their caregivers by providing tailored information, tools, and resources. Patients can leverage their DT to gain insights into their health status, monitor their progress, and make informed decisions about their care. Patients can engage in meaningful conversations about their health and treatment options by sharing the DT data with their healthcare team. This collaborative approach prioritizes the patient’s voice, ensuring their preferences and goals are considered in the decision-making process. Moreover, the data collected from DT can help identify population-level trends, patterns, and insights, which are instrumental in supporting public health initiatives, advancing research studies, and informing policy-making.

Artificial Intelligence needs data to learn from, and development in all its applications to DT is dependent on widespread data collection. Unfortunately, collecting the data required for DT is invasive and disruptive. Educating patient populations about the importance and potential of collecting is critical to realizing the clinical application of DT.

## Data availability statement

Publicly available datasets were analyzed in this study. This data can be found at: https://portal.gdc.cancer.gov/projects/TCGA-BRCA TCGA BRCA dataset.

## Author contributions

H-CC: Investigation, Methodology, Software, Writing – original draft, Writing – review & editing. AG: Data curation, Investigation, Visualization, Writing – review & editing. SK: Data curation, Investigation, Visualization, Writing – review & editing. DW: Writing – review & editing. MS: Data curation, Investigation, Methodology, Project administration, Supervision, Writing – original draft, Writing – review & editing. MM: Conceptualization, Data curation, Investigation, Methodology, Project administration, Software, Supervision, Visualization, Writing – original draft, Writing – review & editing.
